# Soft X-ray Heterogeneous Radiolysis of Pyridine in the Presence of Hydrated Strontium-Hydroxyhectorite and its Monitoring by Near-Ambient Pressure Photoelectron Spectroscopy

**DOI:** 10.1038/s41598-018-24329-8

**Published:** 2018-04-18

**Authors:** Anthony Boucly, François Rochet, Quentin Arnoux, Jean-Jacques Gallet, Fabrice Bournel, Héloïse Tissot, Virginie Marry, Emmanuelle Dubois, Laurent Michot

**Affiliations:** 1 Sorbonne Université, CNRS (UMR 7614), Laboratoire de Chimie Physique Matière et Rayonnement, 4 place Jussieu, 75252 Paris Cedex 05, France; 2 Synchrotron SOLEIL, L’Orme des Merisiers, Saint-Aubin, BP 48, F-91192 Gif-sur-Yvette, France; 3 Sorbonne Université, CNRS (UMR 8234), Physicochimie des Electrolytes et Nanosystèmes Interfaciaux, 4 place Jussieu, 75252 Paris Cedex 05, France; 4 Present Address: KTH Royal Institute of Technology, Department of Applied Physics, Stockholm, Sweden

## Abstract

The heterogeneous radiolysis of organic molecules in clays is a matter of considerable interest in astrochemistry and environmental sciences. However, little is known about the effects of highly ionizing soft X-rays. By combining monochromatized synchrotron source irradiation with *in situ* Near Ambient Pressure X-ray Photoelectron Spectroscopy (in the mbar range), and using the synoptic view encompassing both the gas and condensed phases, we found the water and pyridine pressure conditions under which pyridine is decomposed in the presence of synthetic Sr^2+^-hydroxyhectorite. The formation of a pyridine/water/Sr^2+^ complex, detected from the Sr 3d and N 1s core-level binding energies, likely presents a favorable situation for the radiolytic breaking of the O-H bond of water molecules adsorbed in the clay and the subsequent decomposition of the molecule. However, decomposition stops when the pyridine pressure exceeds a critical value. This observation can be related to a change in the nature of the active radical species with the pyridine loading. This highlights the fact that the destruction of the molecule is not entirely determined by the properties of the host material, but also by the inserted organic species. The physical and chemical causes of the present observations are discussed.

## Introduction

**Figure 1:**
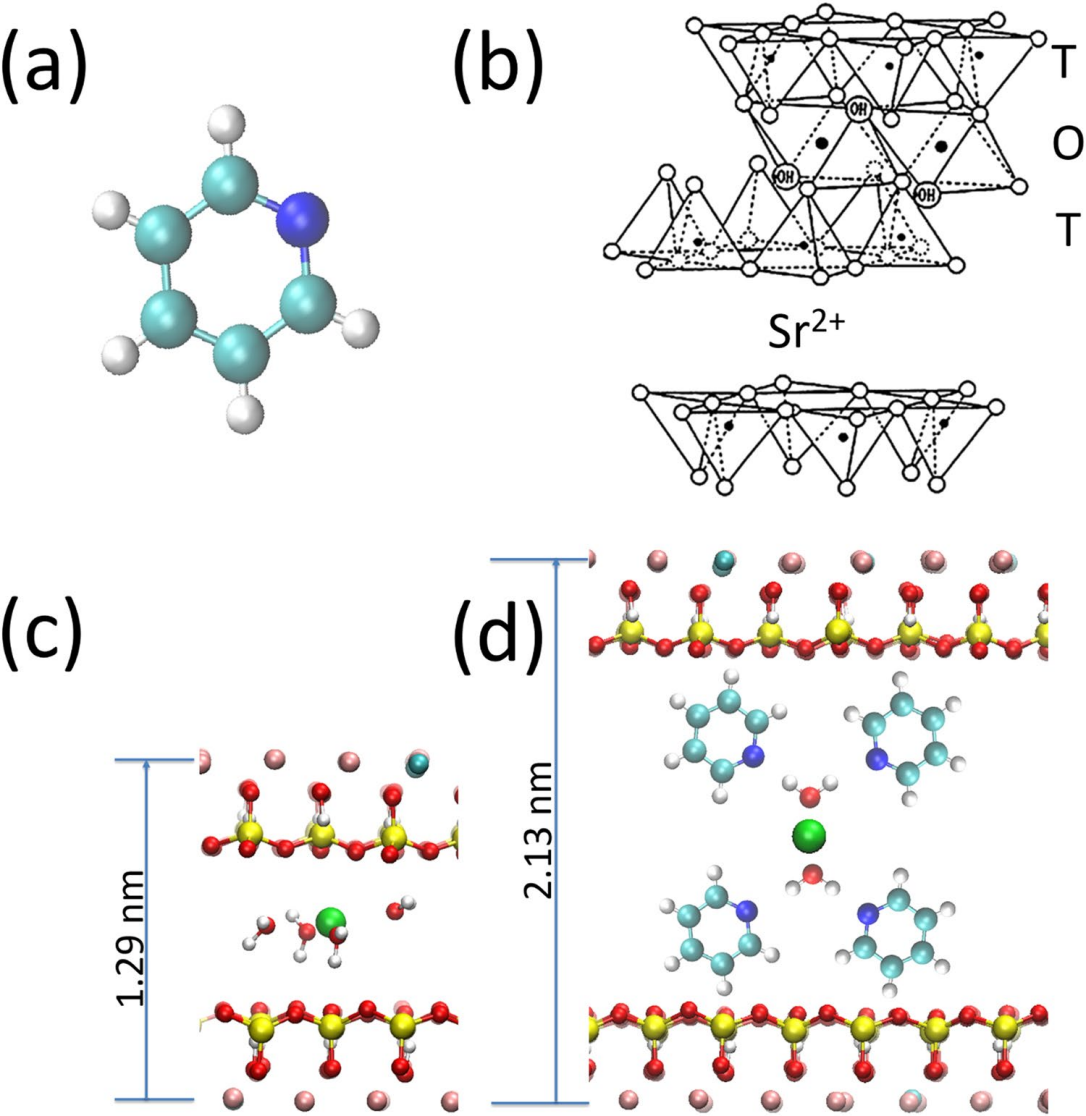
(**a**) The pyridine molecule (nitrogen, carbon and hydrogen atoms are blue, turquoise and white, respectively). The nitrogen atom bears an electron lone-pair, that can make H bonds with water molecules. (**b**) Schematic structure of hydroxyhectorite. The sheet comprises three layers with the TOT arrangement, where T and O designate the tetrahedral silicon oxide layer and the octahedral magnesium oxide layer, respectively. Li^+^ substitutes Mg^2+^ in the O layer, giving a negative charge to the sheet. Sr^2+^ counterions are located in between the phyllosilicate sheets, in the so-called “intersheet region”. (**c**) The 1 W hydration state (strontium, oxygen, hydrogen, silicon, magnesium, lithium and atoms are green, red, white, yellow, pink, and dark turquoise, respectively). (**d**) The model of the pyridine hydrated cation (1 W) complex by Farmer *et al*. (from IR data^5^) and Ukrainczyk *et al*. (from NMR data^7^): note that a water molecule makes two H bonds with two pyridine molecules.

The interaction of organic molecules with clay minerals is a far reaching topic that intersects with catalysis^1–4^, environmental sciences^5–10^, planetary science, astrobiology and astrochemisty^11–19^. Because of the key importance of the interaction between clays and organic matter, we addressed the question of the chemistry of a small organic molecule, pyridine (C_5_H_5_N, Fig. 1(a)), in a lamellar swelling phyllosilicate, hydrated strontium-exchanged hydroxyhectorite (Sr^2+^-hydroxyhectorite), shown in Fig. 1(b). Pyridine is the nitrogen heterocycle analog of benzene, akin to important biomolecules, like pyrimidinic nucleobases. It is also a basic unit of poly-4-vinylpyridine^9^, a polymer we can think of for the recovery of toxic metal complexes after insertion in clays^10^. With respect to its isoelectronic analog, benzene, pyridine is characterized by its nitrogen lone-pair that may form hydrogen bonds with water of hydration^20^. For its part, hydroxyhectorite is a swelling phyllosilicate, present on Earth, Mars^21^ and possibly in carbonaceous chondrites^22^. The alkaline –earth counterion Sr^2+^ has a high water hydration energy that drives the swelling of the smectites^23^. However, it has been so far relatively little associated with hectorite in experimental studies, despite the fact that the ^90^Sr isotope (a β^−^ emitter) is a principal component of many radioactive wastes^24–26^. In the context of nuclear waste confinement, the radiolysis of water in clays leads to the subsequent production of H_2_^27^ and consequently raises serious safety problems. In fact, the present synchrotron radiation study will shed new light on these radiolytic phenomena.

Synchrotron radiation near-ambient pressure X-ray photoelectron spectroscopy (NAP-XPS) in the mbar range was used to study the pyridine/hydrated clay system. The sample environment allows choosing well-defined conditions of relative humidity (RH) and organic molecule vapor pressure. Then we can monitor in real time the influence of the partial pressures of pyridine and water on molecular adsorption, as well as on catalytic reactions in the presence of clay. The control of the partial pressures adds a notable flexibility with respect to other spectroscopic techniques that were previously used to examine the pyridine/clay system^5,7,28–30^. In the latter cases, indeed, samples were prepared by immersion of the clay in pyridine or water/pyridine mixtures, or by saturating the solid with pyridine vapors. Moreover, in the context of heterogeneous catalysis, NAP-XPS has the advantage of providing a synoptic view of the core-level photoelectrons from both the gas phase (reactants and products) and the solid-phase (the surface and adsorbed species). Once the reaction regime changes (e.g. by appearance or disappearance of a gas phase product), the chemistry of the solid surface can be immediately checked^31^.

The present work is original in several aspects. First, the chemistry of hydrated clays had never been studied yet by NAP-XPS, despite many other, environmentally relevant, water/oxide systems were successfully examined by this technique^32^. Consequently, the pyridine/hydroxyhectorite system is definitely a new case for the application of NAP-XPS. Second, the present experiment outreaches the simple question of clay swelling in the presence of pyridine. Indeed, the noticeable phenomenon revealed by this real-time experiment was the steady-state production of N_2_, resulting from the decomposition of pyridine. The latter was induced by the synchrotron radiation soft X-ray photon beam (both intense and highly ionizing), concomitantly with the XPS measurement, in the presence of the clay material. Water confined in clays, and more generally at the surface of oxides, is decomposed into radicals (H, HO) under irradiation by high energy particles, *e*-beams and γ-rays^27,33–36^. Then pyridine reacts with the radicals at the surface of the clay. However, we find that the production of these active species depends on the pyridine partial pressure, emphasizing the fact that the question of the stability of an organic molecule in a clay under irradiation is rather complex. Therefore, our study can contribute to the general issue of the production, conservation or destruction of organic molecules (including biomolecules) in phyllosilicates, a topic of interest for astrochemistry and planetology^12,16–18^, mostly studied under e-beam and γ-ray irradiation. However, soft X-ray irradiation studies are scarce, while they are relevant to such environments as dense molecular clouds and proto-stellar discs, as discussed in the study of solid-state nucleobase degradation under the synchrotron beam^37^. To our knowledge, the soft X-ray radiolysis of organics in the presence of hydrated clays had not received any attention prior to this work. Finally, by highlighting the steady state of a radiocatalytic reaction under given reagent pressures, the present work differs from the previously cited studies, which aimed essentially at determining the lifetime of a molecular species from the decay a known amount of molecules.

Our paper is organized as follows. After recalling how Sr^2+^-hydroxyhectorite swells in the presence of water and pyridine, we examine how the inserted cation core-level binding energy varies due to the adsorption of the molecules. Then the question of pyridine decomposition under the beam is addressed, as manifested by gas- and solid-phase product components appearing in the spectra. Then we discuss possible mechanisms taking into account the band structure of the oxide that aim at explaining the regime change observed (from pyridine mineralization to “protection”) when the pyridine partial pressure overcomes a certain threshold.

## Results and Discussions

### The structure of synthetic Sr^2+^ hydroxyhectorite

Synthetic Sr-exchanged hydroxyhectorite (Sr^2+^-hydroxyhectorite), depicted in Fig. 1(b), is a layered material. The sheets of thickness ~0.75 nm^38^ (including the oxygen radius) comprise two tetrahedral silicon oxide layers sandwiching one central octahedral magnesium oxide layer^39^. Substitution of Mg^2+^ by Li^+^ in the octahedral layer leads to a negatively charged sheet^1^. This charge is compensated by positive ions in the intersheet region, Sr^2+^ in the specific case, leading to a unit cell formula of Sr_0.4_Mg_5.2_Li_0.8_Si_8.0_O_20_(OH)_4_.

The high hydration energy of Sr^2+^ drives the swelling^23,40–42^ of this clay by insertion of water layers, one (1 W), or more, increasing the basal plane spacing, that increases from1.07 nm (dry clay) to1.29 nm (1 W hydration state). The clay film (see Methods) was exposed to a partial pressure of H_2_O (0.5 mbar) at 2 °C, corresponding to a RH of 7%. At this RH, a 1 W hydration state is reached, as in the parent system Ca^2+^-hydroxyhectorite^7,41^ (Ca^2+^ and Sr^2+^ have comparable hydration energies^42^). Keeping the water pressure constant, a partial pressure of pyridine was added, varying from 0.1 to 0.5 mbar.

Due to the deposition process, the clay sheets are mainly parallel to the substrate. Hence, as XPS is a surface sensitive technique, the estimated photoelectron inelastic mean free paths (imfp) are systematically compared to the sheet thicknesses and the basal plane spacing (see below).

### Core-level spectroscopy

**Figure 2:**
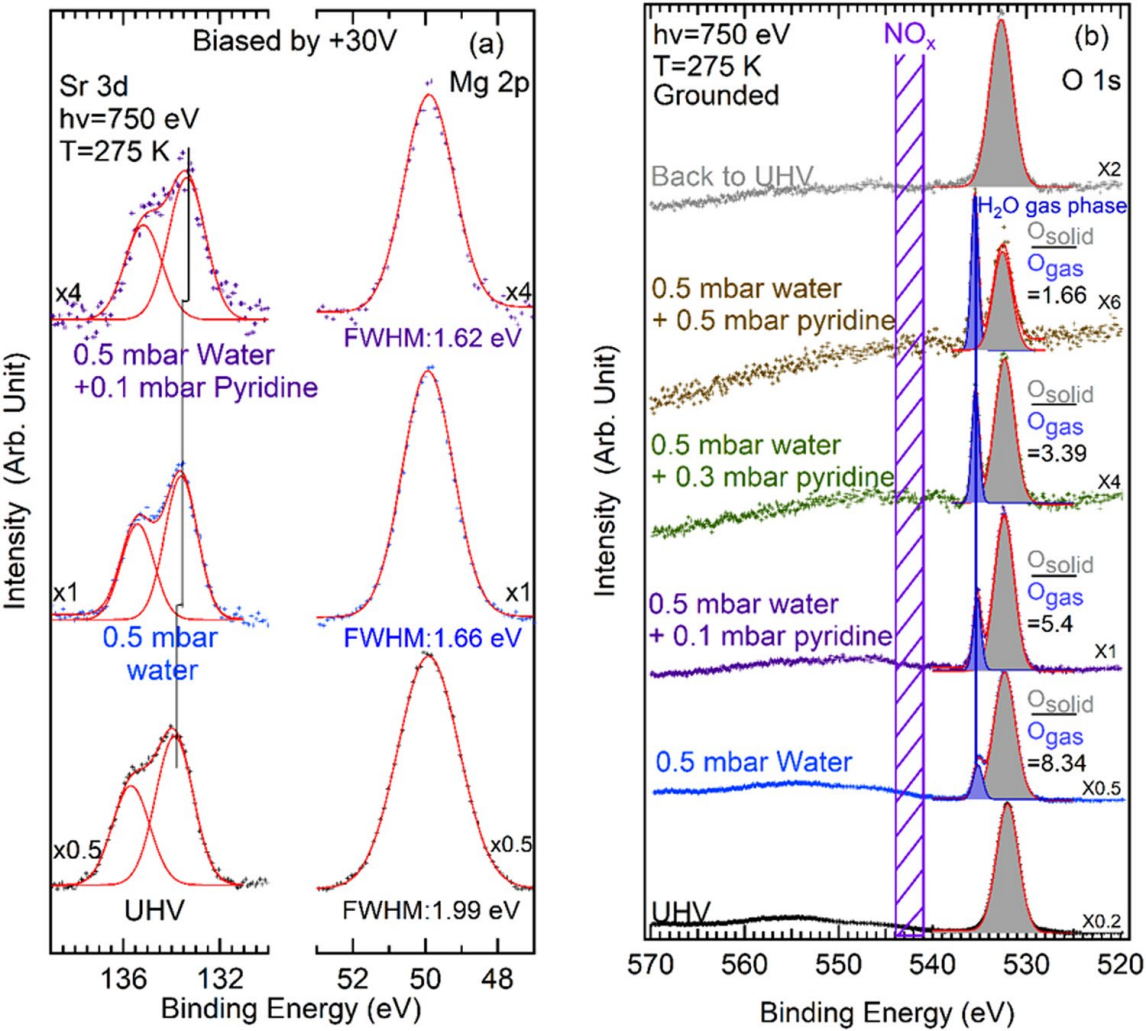
(**a**) Sr 3d and Mg 2p spectra (small crosses) measured at hν = 750 eV with a sample bias of +30 V (the binding energies are corrected). The Sr 3d spectrum is fitted with a 3d_3/2_/3d_5/2_ doublet (red solid line). The Mg 2p level is also a doublet but because of the small spin-orbit splitting energy (0.28 eV), it is fitted by a single component. (**b**) O 1s spectra (small crosses) of the grounded sample measured at hν = 750 eV. Curve fits (red solid line) are also given. The grey component corresponds to the clay lattice oxygen and to confined water, while the blue one corresponds to gas phase water. In all cases, the sample is kept at +2 °C. The water pressure is 0.5 mbar (RH = 7%). Pyridine gas is added (partial pressures are indicated, from 0.1 to 0.5 mbar) while the water pressure is kept constant. All spectra are aligned with respect to the Mg 2p maximum at 50.00 eV and the O 1s maximum (clay) at 532.32 eV (see text and Supporting Information, section S1). We recall to the reader that the gas-phase contribution can only be seen when the sample is grounded (see Methods).

**Figure 3:**
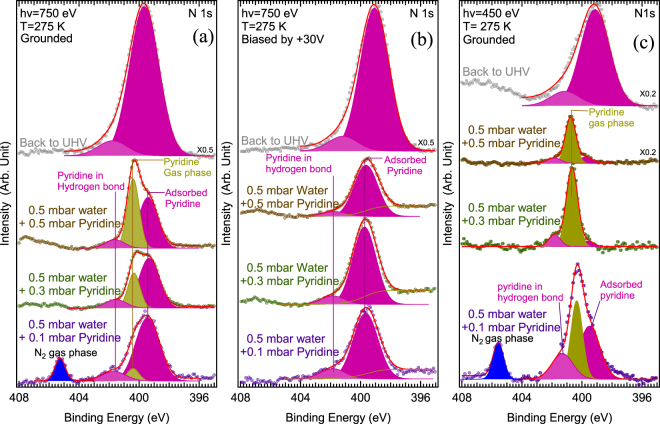
(**a**) N 1s spectra (small crosses) of the grounded sample measured at hν = 750 eV; (**b**) N 1s spectra (small crosses) of the +30 V biased sample measured at hv = 750 eV; (**c**) N 1s spectra of the grounded sample measured at hν = 450 eV (surface sensitive conditions). Curve fits (red solid line) are also given. In all cases, the sample is kept at +2 °C. The water pressure is 0.5 mbar (RH = 7%). Pyridine gas is added (partial pressures are indicated, from 0.1 to 0.5 mbar) while the water pressure is kept constant. After pumping down the gas mixture, a pressure of 10^−7^ mbar is recovered. All spectra are aligned with respect to the Mg 2p maximum at 50.00 eV and the O 1s maximum at 532.32 eV (see text and Supporting Information, section S2). We recall to the reader that the gas-phase contribution can only be seen when the sample is grounded (see Methods).

The core-levels of the clay elements and water are given in Fig. 2 (Sr 3d, Mg 2p and O 1s), and those related to the organic molecule in Figs 3 (N 1s) and 4 (C 1s) respectively. Core-level fitting parameters are reported in the Supporting Information (section S1). All binding energies (BE_FL_) are referenced to the sample Fermi level.

The synoptic view of the gas and condensed phases is obtained when the sample is *grounded*. When the sample is *positively biased* by +30 V, the gas phase contribution is cancelled (see Methods). Differential charging resulting from the insulating nature of the material is also eliminated when the sample is positively biased, and the improvement is particularly noticeable in UHV (see Methods and the Supporting Information, section S2).

### The hydration step

The Sr 3d spectrum of the biased sample measured in ultra-high vacuum (UHV) conditions at hν = 750 eV is shown in Fig. 2 (a) (bottom curve). The estimated^43–45^ imfp is ~1.9 nm for the Sr 3d photoelectrons of kinetic energy ~615 eV and ~2.1 nm for Mg 2p (kinetic energy of 700 eV).

These imfp are very close to the sum of the phyllosilicate sheet thickness (~0.75 nm) and the basal spacing in the dry state (1.07 nm). We recall that 95% of the photoelectrons come from a probed depth equal to 3 × imfp. The Sr 3d spectrum is fitted with a single 3d_3/2_/3d_5/2_ doublet (FWHM = 1.82 eV), with the 3d_5/2_ component positioned at a BE_FL_ of 133.95 eV. This spectral reconstruction suggests a single chemical environment for the strontium ion. In fact, one may have expected that the alkaline earth ions sitting at the surface could be distinguished from those in the intersheet region, due to smaller relaxation energy for the former ones (in a dielectric response scheme^46^, the upper half space is the vacuum) and because more water could be retained in the intersheet space than at the surface. In the Supporting Information, section S3, we give a detailed *hν-*dependent analysis of the Sr 3d spectra, measured in positive bias conditions (to eliminate differential charging). We observe that the FWHM of the Sr 3d doublet increases when the estimated^43–45^ imfp increases (from 1.1 nm to 2.7 nm) for *hν* varying between 450 eV and 1050 eV. However, no chemically-distinct components can be significantly resolved.

When the water pressure is raised to 0.5 mbar (RH = 7%), the Sr 3d_5/2_−Mg 2p energy difference diminishes by ~0.3 eV. The binding energy difference variation is essentially due to a change in the chemical environment of the Sr^2+^ ions. Indeed, Mg ^2+^ ions that nest in the octahedral layer in the middle of the clay sheet are not sensitive to the presence of water. Under exposure to water and formation of a full 1 W hydration state, more water molecules appear in the coordination shell of Sr^2+^ both at the external surface and in the intersheet region. In these conditions, the Sr 3d chemical shift observed upon changing RH from 0% to 7% can be interpreted in terms of either an initial state effect, i.e. a change in the electrostatic energy felt by the strontium atom resulting from swelling (see Supporting Information, section S4), or by a final-state effect, i.e. a change in the dielectric screening due to Sr^2+^ hydration. It must be pointed out that those two effects can also operate jointly^46^.

### The adsorption states of pyridine at 7% RH

When 0.1 mbar of pyridine is added to the water base pressure of 0.5 mbar (7% RH), the Sr 3d_5/2_ − Mg 2p energy difference still diminishes by 0.2 eV (Fig. 2(a)). Consequently, NAP-XPS shows that the Sr^2+^ ion “feels” the presence of the pyridine molecules that penetrate the intersheet region. For still higher pyridine partial pressures (0.3 mbar and above), the Sr 3d signal becomes very weak, almost non-measurable (see Supporting Information, section S5, Figure S4). This originates from two conjugated effects, the attenuation of the photoemission signal (the imfp of the photoelectrons in the gas phase is inversely proportional to the pressure^47^), and the adsorption/sorption of pyridine. The latter phenomenon is clearly demonstrated by the clay component of the (grounded sample) O 1s spectrum (Fig. 2(b)) that remains measurable up to a pyridine pressure of 0.5 mbar. As the O 1s spectra are recorded under constant water pressure, the intensity of the solid (component shaded in gray) can be normalized by dividing it by that of the water vapor (component shaded in blue). The O_solid_/O_gas_ intensity ratio, reported in Fig. 2(b), decreases regularly with increasing pyridine pressure. This means that the phyllosilicate O 1s contribution is significantly damped by pyridine adsorption at the external surface of the layers. Adsorption in the intersheet region must be also considered, as the observed strong damping can be assigned to an additional swelling. Indeed, in the parent compound, Ca^2+^-hydroxyhectorite^7^, the basal plane distance changes from 1.29 nm (1 W) to 2.13 nm when pyridine gets into the intersheet region. Considering the initial basal plane spacing for 1 W Sr^2+^-hydroxyhectorite, the further lattice expansion of ~0.8 nm is comparable to the relatively small imfp of the O 1s photoelectrons, ~0.9 nm^43–45^ at a kinetic energy of ~220 eV (hν = 750 eV).

The core-levels relative to the organic molecule are shown in Figs 3 (N 1s) and 4 (C 1s). The N 1s spectra in Fig. 3(a,b) (measured at hν = 750 eV) and in Fig. 3(c) (measured at hν = 450 eV) give clues about both the chemical state of adsorbed pyridine and its interaction with the clay layers. For N 1s photoelectrons, the imfp in the solid is estimated^43–45^ to be ~1.3 nm at a kinetic energy of ~350 eV (at hν = 750 eV) and 0.8 nm at a kinetic energy of ~50 eV (at hν = 450 eV). Both gas-phase and solid-phase signals are present in the grounded sample spectra of Fig. 3(a). In the spectra measured in biased condition (see Fig. 3(b)), the gas-phase contribution is practically eliminated, appearing as a small background that increases to lower binding energies. By comparing panels 3 (a) and 3 (b), it clearly appears that, under a partial pyridine pressure of 0.1 mbar, the two N 1s components at BE_FL_ of 400.4 eV and 405.35 eV pertain to the gas phase, and that the other two components at 399.5 eV and 401.5 eV pertain to the solid phase.

**Figure 4:**
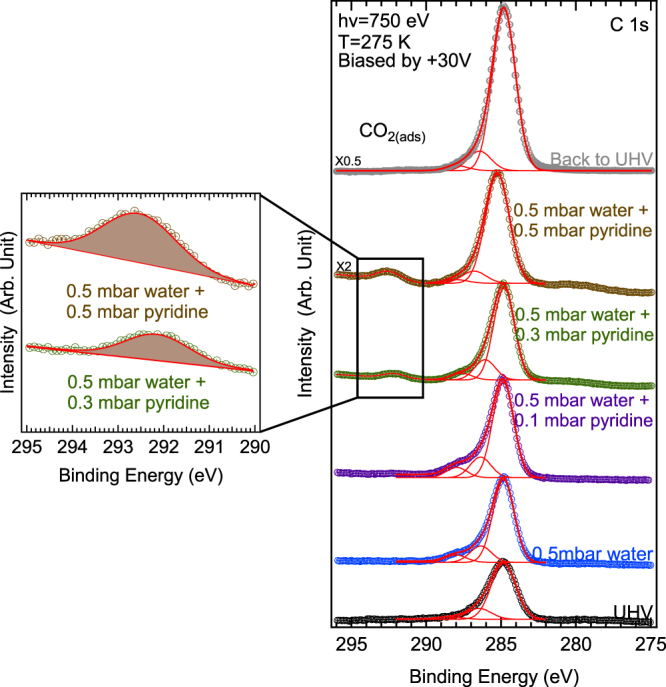
C 1s spectra measured at hv = 750 eV (circles) while the sample is biased to +30 V. Curve fits (red solid lines) are also given. In all cases, the sample is kept at +2 °C. The water pressure is 0.5 mbar (RH = 7%). Pyridine gas is added (partial pressures are indicated, from 0.1 to 0.5 mbar) while the water pressure is kept constant. The UHV pressure is 10^−8^ mbar. After pumping down the gas mixture, a pressure of 10^−7^ mbar is recovered. All spectra are aligned with respect to the Mg 2p maximum at 50.00 eV and the O 1s maximum at 532.32 eV (see text and Supporting Information, section S2). We recall to the reader that in biased sample conditions the gas phase contribution is eliminated (see Methods).

We now focus on the two solid-phase components in the N 1s spectra. The low binding energy component at 399.5 eV is attributed to adsorbed pyridine molecules with free N lone-pairs, i.e. that are not engaged in dative bonding^48,49^ or in hydrogen bonding^50–54^. For its part, the high binding energy component at 401.5 eV is attributed to pyridine molecules engaged in hydrogen bonds (H-bonds) with adsorbed water molecules. Pyridine, a Lewis base, acts as a H-acceptor. The effect on the N 1s binding energy is essentially electrostatic in nature^53^, inducing a shift to higher binding energy with respect to the free lone-pair component at 399.5 eV. This is illustrated by the specific case of isonicotinic acid, a pyridine carboxylic acid, for which a sizeable binding energy shift of 1.7 eV is observed between “non hydrogen-bonded” nitrogen atoms and nitrogen atoms in acceptor O−H···N bonds^52^. In the parent compound, 1 W Ca^2+^-hydroxyhectorite, NMR^7^ detects strong signals due to pyridine making no H bonds, attributed to mobile intercalated pyridine and pyridine physisorbed on the outer surface, or pores. NMR^7^ also detects pyridine forming H bonds with the solvation shell of the cation. In Fig. 1(d) we give a possible model of H-bonded pyridine in 1 W Sr^2+^-hydroxyhectorite, inspired by the infrared spectroscopy work of Farmer *et al*.^5^ for Ca^2+^ complexes. Four pyridine molecules are placed around the first hydration shell of the cation, making acceptor H-bond with water. The molecular plane is orthogonal to the phyllosilicate plane, and the molecular C_2_ axis makes an angle of 45° with the latter.

The presence of a pyridinium signal in the N 1s spectra (Fig. 3) is excluded, because the 2 eV binding energy difference observed between the two solid-phase components is significantly smaller than that measured between neutral pyridine and the pyridinium ion (about 2.65 eV^55^). It is worth noticing that in Ca^2+^-hydroxyhectorite, the pyridinium NMR signal is within the detection limit^7^. Due to the bigger size of their counterions, hydrated Sr^2+^-hydroxyhectorite, together with Ca^2+^-hydroxyhectorite, are thus much less acidic than Mg^2+^ exchanged smectites^5,56^ where IR spectroscopy indicates that the proton is transferred to the pyridine molecule.

The “non H-bond” to “H-bond” to intensity ratio is ~6.6 at *hν* = 750 eV under 0.1 mbar of pyridine (Fig. 3(b)). At this excitation energy the estimated imfp is ~1.3 nm in the clay. More surface sensitive conditions are reached at *hν* = 450 eV (Fig. 3(c)). Then the imfp in the clay is minimal, ~0.8 nm^43,44,57^, nearly a factor of two smaller than the imfp at *hν* = 750 eV and practically equal to the phyllosilicate sheet thickness (~0.75 nm). At *hν* = 450 eV, the “non H-bond” to “H-bond” intensity ratio decreases to 2.1 (under a pyridine partial pressure of 0.1 mbar for which a reliable component weighing is doable, as the gas pressure is not too high, see Methods). The fact that the “non H-bonded” pyridine weight is smaller at the external surface of the clay can be tentatively explained by considering that the absolute value of the physisorption energy is greater when the molecule is confined between two phyllosilicate sheets than when it is adsorbed on the outer surface.

In the Supporting Information, section S5, we estimate the *maximum* “non H-bonded” to “H-bonded” pyridine ratio in the intersheet region. We find a ratio of ~5. This value compares with the ratio measured in bulk sensitive conditions, but it is notably greater than that measured in surface sensitive ones. “Non H-bonded” molecules prefer to adsorb in the interstice between the sheets rather than on the outer surface.

### The mineralization of pyridine

We now consider the gas-phase components at BE_FL_ of 400.4 eV and 405.35 eV in Fig. 3(a) (grounded sample). These two components arise from core-ionized molecules present in the gas volume in contact with the solid. The vacuum level of the molecules is pinned to the vacuum level of the solid, and thus BE_FL_ is simply the difference between the gas phase ionization energy referenced to the vacuum level (IE_VL_) and the solid work function, neglecting the gradient of the contact potential difference qV_cpd_ between sample and analyzer (see Methods). The lower energy component (BE_FL_ of 400.4 eV) that increases with pyridine partial pressure is ascribed to gas-phase pyridine. As the N 1s IE_VL_ of pyridine 404.9 eV^58^, the other gas phase component seen at higher binding when the pyridine partial pressure is 0.1 mbar, corresponds to an IE_VL_ of 409.95 eV. This is precisely that of dinitrogen (N_2_)^58^. Gaseous NO is excluded. NO being a radical, it should exhibit a N 1s doublet at IE_VL_ 410.1 and 411.5 eV (i.e. at BE_FL_ of 405.5 eV and 406.9 eV) and an O 1s doublet at IE_VL_ = 543.2 and 543.8 eV^58^ (i.e. at a BE_FL_ of 538.6 and 539.2 eV), observed neither in the N 1s window (Fig. 3(a)) nor in the O 1s one (Fig. 2(b)). HCN (IE_VL_ of 406.15 eV, and BE_FL_ of 401.65 eV) and NH_3_ (IE_VL_ of 405.6 eV, and BE_FL_ of 401.1 eV), if present, would merge with the H-bonded pyridine component at BE_FL_ of 401.5 eV. The comparison of the fitted curves (grounded versus biased) shows that the “non H-bonded” to “H-bonded” pyridine intensity ratio is not affected (Fig. 3(a,b)). Therefore, there is no indication of the presence of NH_3_ and HCN molecules in the gas phase.

When the partial pressure of pyridine reaches 0.3 mbar, the production of gaseous N_2_ stops, as shown in Fig. 3(a). Concomitantly, see Fig. 4, a new component starts to grow in the C 1s spectrum, at a BE_FL_ of 292.15 eV (*hν* = 750 eV, imfp ~ 1.5 nm^43–45^). As the sample is* positively biased*, this component must be attributed to a *solid-phase* species. The signal still increases when the pyridine partial pressure reaches 0.5 mbar. However, when the gas mixture is pumped down, the component disappears from the C 1s window, showing that the species is weakly bonded. The observed C 1s BE_FL_ of this labile species corresponds precisely to that of CO_2_ physisorbed on oxide surfaces (291.8 eV^59^). This attribution to *adsorbed* CO_2_ is reasonable, especially since the BE_FL_ is significantly higher than that of *strongly bound* carbonates (289.0–289.6 eV) or hydroxycarbonates (290 eV)^60^.

The identification of both N_2_ in the gas phase (under a pyridine partial pressure of 0.1 mbar) and adsorbed CO_2_ (in the 0.3–0.5 mbar range) provides a clear indication of the mineralization of pyridine. As the sample temperature of +2 °C is far below the range where thermally activated reactions in hectorite are expected to occur^2^, this phenomenon must be linked to the interaction between the synchrotron X-ray beam and the solid sample. The N_2_ yield is certainly not due to a gas phase reaction, as it is nil when the pyridine partial pressure reaches 0.3 mbar. Moreover, we have observed that the N_2_ yield under a pyridine partial pressure of 0.1 mbar depends on the irradiation conditions, i.e. the photon energy and/or the photon flux (×2 between 450 eV and 750 eV, see Methods). The intensity ratio $\frac{gaseous\,N2\,peak\,}{gaseous\,pyridine\,peak}$ is 0.21 at *hν = *450 eV (Fig. 3(c)) and increases to 1.57 at *hν* = 750 eV (Fig. 3(a)), i.e. by a factor of 7.5.

Hectorite is an insulator with a band gap of ~ 4.0–4.5 eV^61^. In this material, photoelectrons and Auger electrons produced by X-ray irradiation lose energy during *their transport* in the insulating material by creating valence electron-hole (*eh*) pairs^62^. In turn, electrons and holes react with water, leading to the formation of radical species^27,34–36^, that degrade the pyridine molecule. The number of *eh* pairs produced by one (absorbed) photon of energy *hν* and having ionized a given core-level is approximately *hν*/*E(eh*), where *E(eh*) is the energy for creating a *eh* pair, is two to three times the bandgap. Taking into account the *hν-*dependent overall ionization cross-sections for the clay compound of formula Sr_0.4_Mg_5.2_Li_0.8_Si_8.0_O_20_(OH)_4_ (Supporting Information, section S6), the hole-electron pair generation factor per unit volume and per unit time *g*(*eh*) (proportional to *hν*, the photon flux and the linear absorption coefficient^62^) increases by a factor ×3.4 between 450 eV and 750 eV (Supporting Information, section S6), a value that compares well with the observed increase (×7.5) in the N_2_ yield. Auger final states in oxides^62^ and in confined water^63,64^ above the O 1s edge (~530 eV) may also play a role in beam damage, but the present estimates of *g*(*eh*) show this is not a dominating factor.

### Radiolytic reaction schemes

The radiolysis of pyridine was extensively studied in “bulk” aqueous solutions, using the pulsed e-beam technique, combined with UV-visible absorption spectroscopy^65–67^. Solvated electrons, H^•^ and HO^•^ radicals resulting from the dissociation of water react with pyridine to give pyridinyl radicals, H and HO adducts. These experiments give unique information on the kinetics of the primary species (e.g. the reaction rates of the solvated electron)^67^. However they do not point to dissociation products, in contrast to the present case. The reason for this is a question of dose. In fact while dose rates are huge in both case, ~10^9^ Gy/s for a pulsed e-beam experiment^67^ and ~10^7^ Gy/s for NAP-XPS (see the Supporting Information, section S7), the overall doses differ considerably, 6 Gy per pulse in the former case^67^ (6 pulses are sufficient to acquire an absorption spectrum) and more than 10^9^ Gy in the latter case (considering that the minimum acquisition time is 25 s).

To our knowledge, there is no account of the radiolysis of pyridine adsorbed onto a hydrated smectites using the pulsed e-beam technique. However, admitting, as in bulk water^67^, that the primary products of water radiolysis subsequently react with pyridine, e-beam studies of the radiolysis of confined water in hydrated smectites are quite relevant to the present case^35,36^. The radiolysis of water in clays presents specific traits with respect to the bulk water case. First, the transfer of energy from the oxide (where *eh* pairs are produced as discussed before) to water seems to be facilitated by confinement. Indeed, in hydrated montmorillonite and saponite, the H_2_ yield is highly enhanced for the 1 W hydration state, while it is practically equal to that of bulk water in the 2 W state. Second, impurities in the clay sheets (such as the presence of Fe^3+^) are quite efficient to quench the H_2_ production as electron trapping sites^36^. Although such structural ions are absent in the present synthetic clay, Ref.^36^ suggests that electron scavenging chemical species (i.e. the pyridine molecule itself, see below) may play a crucial role.

As already stated, we observe here two regimes under soft X-ray irradiation, the steady-state production of N_2_, under a pyridine partial pressure of 0.1 mbar, and the formation of CO_2_ adsorbates under higher pressures, with no gaseous N_2_ being produced anymore. The first regime can be ascribed to a hydrogenolysis/denitrogenation process, while the second one can be attributed to an oxidation one. This strongly suggests that the reactive radicals are different in each case, and that their nature depends on the pyridine partial pressure.

Pyridine dissociation to N_2_ requires breaking the aromatic ring. As no NO_x_ products are observed, we consider primarily the role played by the H^•^ radical. Both the abstraction of a pyridine H atom by the H^•^ radical, and its addition to the ring can be envisaged, but the latter is favored over the former, at least in the gas phase^68^. In a hydrogenolysis/denitrogenation scheme, the pyridine ring is first fully hydrogenated by H^•^, forming a piperidine molecule. Hydrogenolysis leads to *n*-pentyl amine (ring opening) and then C_5_ hydrocarbons plus ammonia. C 1s components of gaseous hydrocarbons cannot be identified as their IE_VL_ are close to the m- and p-carbon C 1s IE_VL_ of gaseous pyridine (see Supporting Information, section S8, Figure S5). Analogously, C_x_H_y_ products adsorbed in the clay (solid phase) cannot be distinguished from the main adsorbed pyridine component at BE_FL_ ~284.8 eV (Fig. 4). Gaseous ammonia is not detected in the N 1s spectrum, and N_2_ is observed instead. This would imply that ammonia is decomposed radiolytically^69,70^ in the clay to yield N_2_ and H_2_ (we stress that H_2_ is not detectable by XPS due to the very low H 1s photoionization cross section). The source of H^•^ is the reaction of hydration of the intersheet water with electrons according to:

1 $$e+{{\rm{H}}}_{2}{\rm{O}}\to {{\rm{HO}}}^{-}+{{\rm{H}}}^{\bullet }$$

This dissociation would be all the easier^36^ as we reached the state 1 W with a RH of 7%.

**Figure 5:**
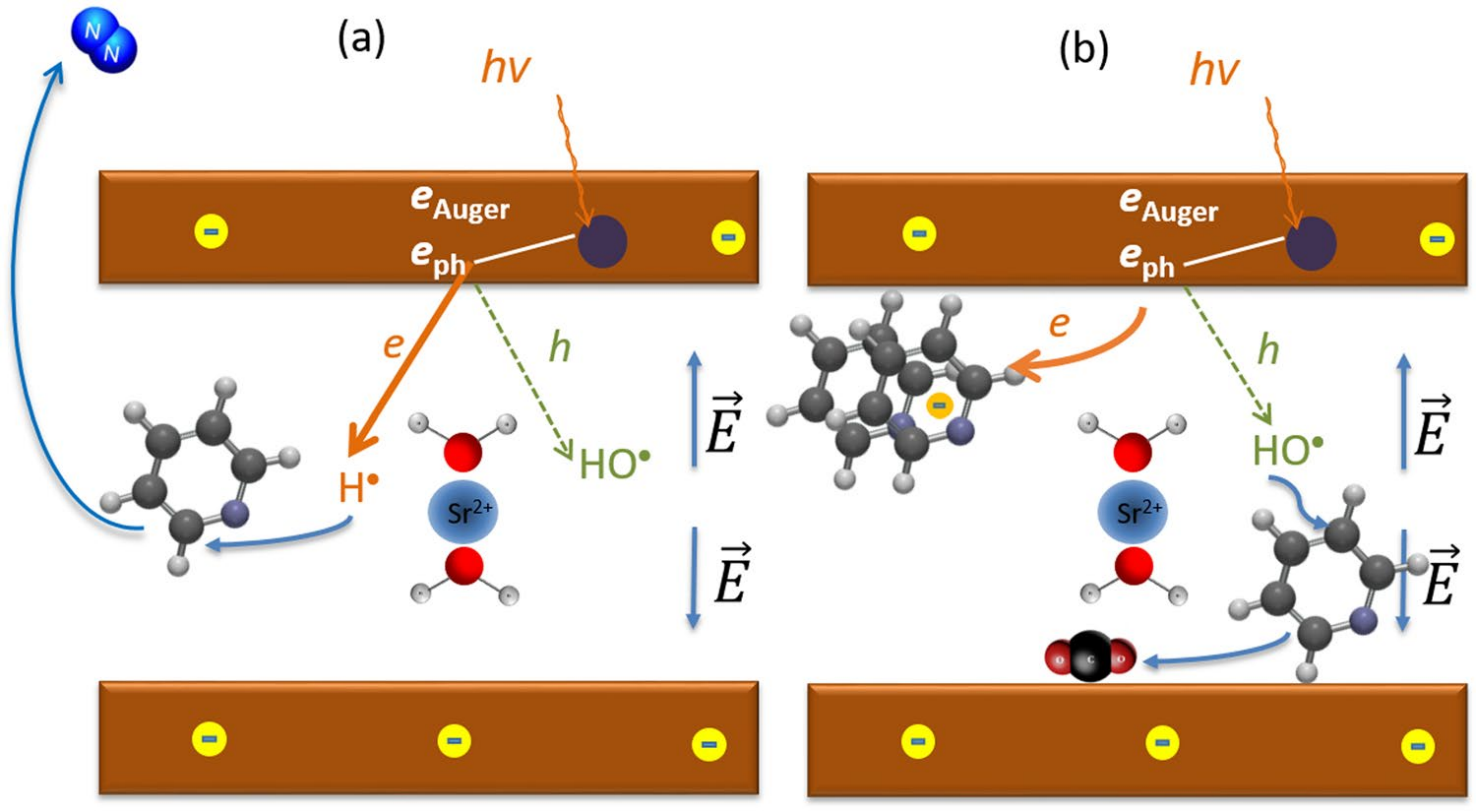
Radiolysis schemes, (**a**) corresponding to the production of gaseous N_2_ (pyridine partial pressure of 0.1 mbar) and (**b**) to the production of adsorbed CO_2_ (pyridine partial pressure of 0.3 mbar and 0.5 mbar). e_ph_ and e_Auger_ are photoelectrons and Auger electrons, respectively, produced under X-ray irradiation (hν). e and h are valence electrons and holes, respectively, created as e_ph_ and e_Auger_ lose energy in the oxide. $\overrightarrow{E}$ is the electric field.

The electrostatic field (the phyllosilicate layer is negatively charged and the cations are in the intersheet region) should facilitate the injection of electrons in the intersheet region and block the holes, as depicted in Fig. 5(a). Indeed, we estimate that the electrostatic potential energy variation between the center of the phyllosilicate sheet and the cation plane is in the range −1 to −2.8 eV (see Supporting Information, section S4). Therefore one could consider that the production of H^•^ dominates that of HO^•^, the latter being due to the reaction of a hole with water according to:

2 $$h+{{\rm{H}}}_{2}{\rm{O}}\to {{\rm{H}}{\rm{O}}}^{\bullet }+{{\rm{H}}}^{+}$$

HO^•^ is a strongly oxidizing species. However, the more difficult injection of holes in the intersheet region, and their recombination with excess electrons when they reach this place, may explain why, under 0.1 mbar of pyridine, the inserted species are not oxidized (HO^•^ can indeed add up to pyridine^66,71^) and why no NO_x_ are seen in the gas phase.

It is particularly noteworthy that the steady-state N_2_ production stops when the pyridine partial pressure is raised to 0.3 mbar. We can interpret this pressure effect as due to a strongly reduced production of H^•^. An increasing pyridine loading, as observed in Fig. 2 through the decrease of the *O*_*solid*_ to *O*_*gas*_ ratio, may lead to the blocking of the reaction sites where the radicals are produced from water molecules.

Alternately, pyridine could scavenge the electrons in the hydrated clay. This hypothesis is illustrated in Fig. 5(b). Although pyridine has a negative adiabatic electron affinity in the gas phase, the pyridine cluster anions have a positive affinity that increases incrementally with the number of molecules (up to 0.8 eV for (C_6_H_5_N)_8_^−^)^72^. The same trend is observed for pyrimidine bases embedded in water clusters^73^. Generally speaking, the pyridine radical anion is stable in solution, due to the large solvation energy difference between the negative and the neutral species^74^, and radiolysis experiments report that pyridine in water behaves as an electron scavenger^66,67^. An increased pyridine loading could therefore increase the electron scavenging capacity of the molecule. If such a scheme is correct, electron scavenging diminishes the *e-h* recombination probability, more holes are now available and therefore the yield of HO^•^ radicals via reaction (2) increases. The formation of HO^•^ explains why adsorbed CO_2_ is observed in the C 1s spectrum, with an intensity increasing with pyridine pressure. CO_2_ could result from the oxidation of adsorbed C_x_H_y_ species remaining from the denitrogenation reaction discussed above.

## Summary and Perspectives

In conditions of high flux (~10^16^ photons × cm^−2^ × s^−1^) and high ionization efficiency (the attenuation length is ~ 600 nm in the clay in the 450 to 750 eV range), the soft X-ray monochromatic irradiation of pyridine in the presence of hydrated (1 W) Sr^2+^ hydroxyhectorite can induce the heterogeneous radiolysis of the organic molecule, leading to the steady-state production of N_2_. The chemical bonding of pyridine adsorbed at the outer clay sheets and in the interstice between the sheets is a part of the explanation for the observed radiolysis. The analysis of the XPS binding energies (Sr 3d and N 1s) shows indeed that a hydration shell forms around the cation, and that a sizeable fraction of the pyridine molecules makes a H-bond with the adsorbed water molecules. The formation of preponderant H^•^ radicals, in the immediate vicinity of the H-bonded pyridine, is a likely explanation of the observed steady-state mineralization of the organic molecule to N_2_, under a pyridine partial pressure of 0.1 mbar. When the pyridine partial pressure rises to 0.3 mbar, the N_2_ production stops, and a labile adsorbed CO_2_ species appears instead. This effect is probably due to a halt in the H^•^ yield (that lead to denitrogenation). A reaction site blocking can be thought of. Alternately, one can envisage that the electron scavenging capacity of pyridine increases with the size of the molecular cluster in the clay, which in turn leads HO^•^ production and oxidation.

Bond breaking in water molecules confined to the surfaces of oxides is a primary step in the radiolytic process, about which there is a general agreement. However, at a fundamental level, many questions remain open in the specific case of swelling clays. First, the effect of the electric field present in charged phyllosilicates in separating holes from electrons, and the consequences on the production of active radicals from sorbed water, needs certainly more attention. Second, the fact that radiolytic phenomena in hydroxyhectorite do not only depend on the host material, but also on the pyridine load (via the pyridine pressure), has important consequences for the measurements of half-lives of organic species in clays where spatial conditions are simulated. It is clear that the electron scavenging hypothesis put forward here should be also tested by using organic molecules of the same family, including pyrimidine nucleobases. Indeed, the application of electron scavenging molecular additives in clays for reducing H_2_ yield is also worth exploring.

## Methods

### Materials

The clay mineral was deposited as micrometric layers on a gold-coated silicon wafer. Deposition was carried out by evaporating a dilute aqueous suspension of synthetic hydoxyhectorite^41^. The preparation of the clay dispersion solution used for drop-casting is available in ref.^75^. The hydroxyhectorite platelets have typical dimensions of ~1−2 μm, as determined by atomic force microscopy.

### The NAP-XPS setup at SOLEIL synchrotron facility

The NAP-XPS experiment was carried out at the French synchrotron facility SOLEIL (TEMPO beamline) using the new experimental setup operated by the LCPMR team. The sample is kept at 2 °C using a Peltier cooler. The water pressure was maintained at 0.5 mbar (RH = 7%), and the pyridine pressure was varied between 0.1 and 0.5 mbar. These pressures were below the saturation vapor pressures of water and pyridine (both 7 mbar at 2 °C), and therefore, the liquid phases were not condensed on the sample. The gases are introduced via leak valves into the analysis/reaction vessel, that is itself pumped out via the analyzer nozzle and the windowless beamline entrance. A steady-state regime is reached for which the pressure remains constant.

The synchrotron beam was directed to the sample via a windowless, differentially-pumped entrance, designed by SPECS. The X-ray beam makes an angle of 54° with respect to the analyzer axis. The beamline monochromator exit slit was set to 50 μm, yielding a photon energy resolution $\frac{h\nu }{{\rm{\Delta }}h\nu }$ of ~5000. The inelastic mean free path (imfp) of the photoelectrons in the clay (and hence the probed material thickness) depends on their kinetic energy^43–45^. Therefore different excitation energies *hν* (450, 750 and 1050 eV) were used to vary the probed depth (the estimated imfp are given in the text). The calculated photoionization cross-sections of the constituent atoms are given in the Supporting Information section S5.

In UHV conditions, 1.5 10^12^ photons × s^−1^, and 3 10^12^ photons × s^−1^ and 2 10^12^ photons × s^−1^ reached the sample at 450, 750 and 1050 eV respectively. With a circular X-ray spot area of 8 × 10^−3^ mm^2^, the photon flux was ~2 × 10^16^ photons × cm^−2^ × s^−1^ at 450 eV, ~4 × 10^16^ photons × cm^−2^ × s^−1^ at 750 eV, and ~2.5 × 10^16^ photons × cm^−2^ × s^−1^ at 1050 eV. Along its way across the gas phase (about 5 cm), the photon flux loss due to absorption was almost negligible in the present pressure conditions. Under a pressure of 0.5 mbar of water and 0.1 mbar of pyridine, one estimates that 97%, 98% and 99% of the photons reach the sample surface at photon energies of 450, 750 and 1050 eV, respectively^76^. Typical soft X-ray photon fluxes in space considered in ref.^37^ are orders of magnitude smaller than the present fluxes. They can reach 10^10^ photons × cm^−2^ × s^−1^ in the X-ray dominated photodissociation regions of molecular clouds, but are much smaller in protoplanetary disks, 10^3^ photons × cm^−2^ × s^−1^.

Irradiation doses are discussed extensively in the Supporting Information, section S7 where the absorption coefficient μ is calculated. The latter is ~16,000 cm^−1^ at *hν = *750 eV, corresponding to a characteristic length 1/μ of ~600 nm. The dose rate $\dot{D}$ is ~3 × 10^7^ Gy/s at *hν* = 750 eV. The minimum *D* in the present case is 7.5 × 10^8^ Gy at *hν = *750 eV considering that the acquisition time of one XPS spectrum is 25 s.

The analyzer is a PHOIBOS NAP 150 manufactured by SPECS. The spectra were measured at a pass-energy of 50 V with a slit of 3 mm × 20 mm, corresponding to a calculated analyzer resolution of 500 meV. The NAP-XPS nozzle aperture (of diameter 0.3 mm) was brought close to the sample surface at a short distance of ~1.5 mm to minimize the photoelectron inelastic scattering in the gas phase. All XPS peaks were fitted with Gaussian functions. The Sr 3d core level is actually a “3d_3/2_ 3d_5/2_” doublet, with a spin-orbit splitting energy of 1.8 eV and a branching ratio 3d_3/2_:3d_5/2_ of 0.66 eV. The Mg 2p peak is also a doublet (“2p_1/2_ 2p_3/2_”) but due to its small spin-orbit splitting (0.28 eV) the spectrum is fitted with a single component. This peak can be used as an internal binding energy reference.

### Differential charging and its elimination

Because of their low electrical conductivity, clays charge positively under X-ray photon irradiation. Charging is not uniform, laterally and vertically, and therefore the core-levels may appear broad and distorted (see section S2 of the supporting information). Differential charging, a major issue when minerals are studied, plagues the chemical interpretation of XPS spectra by introducing meaningless components. Differential charging is efficiently alleviated when more negatively charged species (electron and anions) reach the surface. NAP conditions are very beneficial, typically when the gas pressure is above 1 mbar^77^. However below this pressure threshold (and especially in UHV conditions) charging effects remain very critical. We have found that charge compensation is very effective in the 10^−8^ mbar range when the substrate is biased positively (+30 V). Even in NAP conditions, a positive biasing still improves charge compensation. More details can be found in the Supporting Information, where we indicate how charging can be practically suppressed by this original procedure. In some instances, a + 30 V biasing cannot be implemented: for N 1s spectra measured at *hν* = 450 eV, the kinetic energy of the photoelectrons (~50 eV) is reduced to ~20 eV, that is too low to be measured at a pass energy of 50 V.

### Gas-phase versus solid-phase spectral components

**Figure 6:**
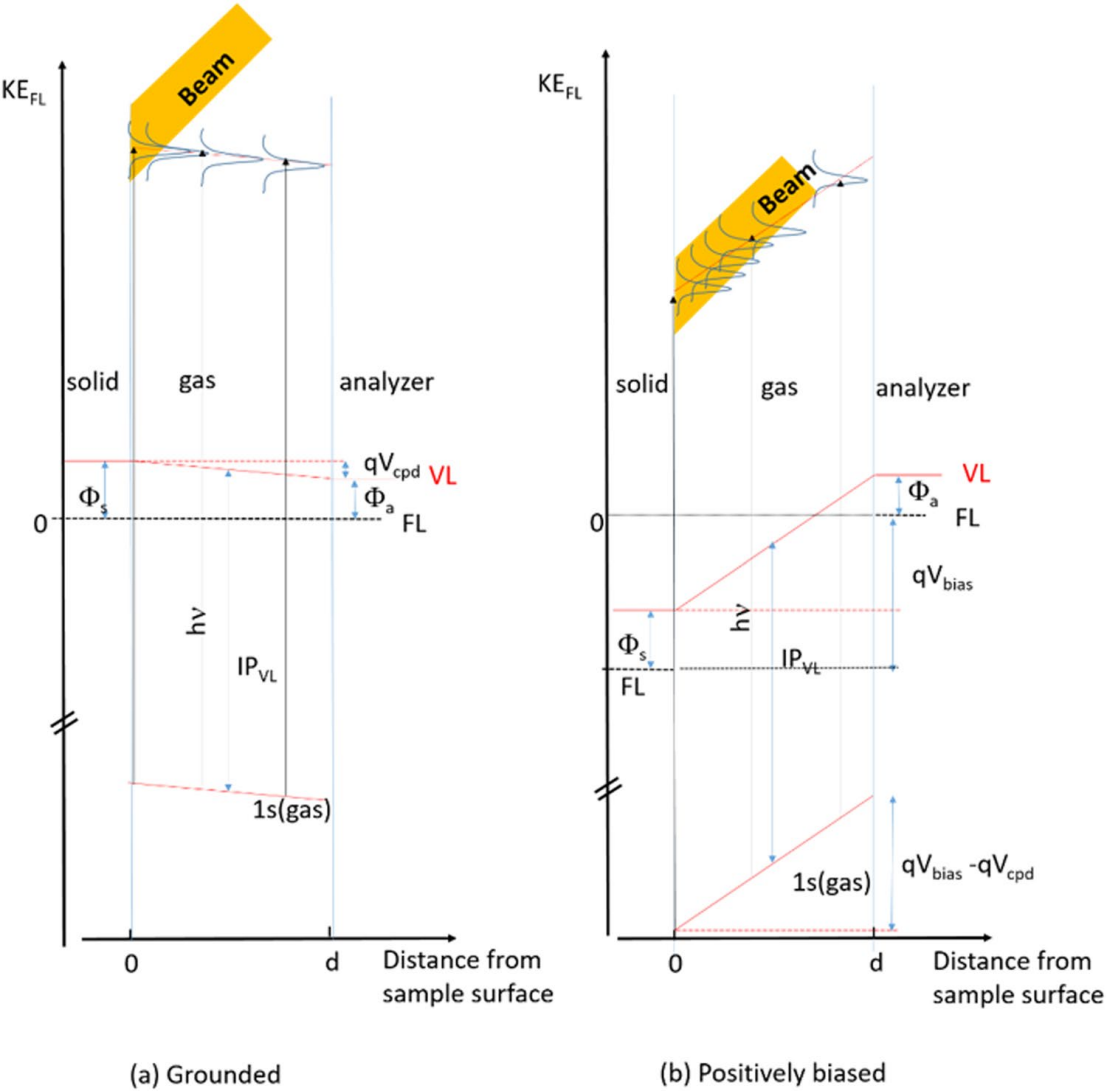
Energy diagrams of the solid phase (sample)/gas phase/analyzer system, when the sample and analyzer are grounded (**a**) and when the sample is biased positively with respect to the analyzer (V_bias_ = + 30 V). d (~1 mm) is the distance between the sample and the analyzer nozzle. VL and FL designate the vacuum and Fermi level respectively. Φ_s_ and Φ_a_ are the sample and analyzer work functions, respectively. 1s(gas) is the core-level of a gas phase molecule, of ionization energy IP_VL_ (with respect to VL). KE_FL_ is the kinetic energy of the 1s(gas) photoelectron measured with respect to the analyzer FL. qV_cpd_ is the contact potential between the sample and the analyzer (equal to Φ_s_-Φ_a_, a few eV), and qV_bias_ the applied electrostatic potential energy (−30 eV). While the gas phase spectrum is measured in a potential gradient qV_cpd_/d of a few eV per mm when the sample is grounded, it is measured in a potential gradient of (qV_bias_− qV_cpd_)/d (~qV_bias_/d), i.e. ~30 eV per mm when the sample is biased. This leads to the spreading of the gas phase 1s core level (1s(gas)) KE_FL_ in the beam-probed region (yellow-shaded) of width ~0.1 mm.

Biasing the sample has also an enormous advantage as it enables distinguishing gas-phase from solid-phase components. The principles of the method are shown in Fig. 6. Solid-phase species (clay and adsorbed species) see their core-level binding energies follow the polarization of the sample and move down by |qV_bias_| (when the positive + V_bias_ potential is applied). On the other hand, the gas-phase species will experiment an apparent contact potential energy difference of about 30 eV downward, between the sample surface and the analyzer nozzle (that is grounded). The gradient is about 30 eV/mm. As the gas-phase region probed by the beam is about 0.1 mm, the gas-phase 1s core-level spectra are spread at least over ~3 eV. The result is the quasi-elimination of the gas-phase contribution that remains as a small background at binding energies lower (at kinetic energies higher) than the solid-phase signal.

## Electronic supplementary material

Supporting Information
