# Supplementary material for: Soft X-ray Heterogeneous Radiolysis of Pyridine in the Presence of Hydrated Strontium-Hydroxyhectorite and its Monitoring by Near-Ambient Pressure Photoelectron Spectroscopy
Source: Sci Rep. 2018 Apr 18;8:6164. doi: 10.1038/s41598-018-24329-8 (PMC5906652; doi:10.1038/s41598-018-24329-8)
Supplement: Supplementary file 1 — Supporting Information [file 41598_2018_24329_MOESM1_ESM.pdf]

Supporting Information

Soft X-ray Heterogeneous Radiolysis of Pyridine in the Presence of Hydrated Strontium-Hydroxyhectorite and its Monitoring by Near-Ambient Pressure Photoelectron Spectroscopy

Anthony Boucly,[1,2*] *François Rochet,*[1,2,3*] *Quentin Arnoux,*[1,2] *Jean-Jacques Gallet,* [1,2,3] *Fabrice Bournel,* [1,2,3] *Héloïse Tissot,* [1,2,3†] *Virginie Marry,*[4,5] *Emmanuelle Dubois,*[4,5] *and Laurent Michot*[4,5]

[1] *Sorbonne Universités, UPMC Univ. Paris 06, Laboratoire de Chimie Physique Matière et Rayonnement (LCPMR), F-75005 Paris, France.*

[2] *CNRS, UMR 7614, LCPMR, 75005 Paris, France*

[3] *Synchrotron SOLEIL, L'Orme des Merisiers, Saint-Aubin, BP 48, F-91192 Gif-sur-Yvette, France.*

[4] *Sorbonne Universités, UPMC Univ. Paris 06, Physicochimie des Electrolytes et Nanosystèmes Interfaciaux (PHENIX), F-75005 Paris, France.*

[5] *CNRS, UMR 8234, PHENIX, 75005 Paris, France*

AUTHOR INFORMATION

***Corresponding Authors** francois.rochet@upmc.fr; boucly.anthony@gmail.com

[†] *Now at KTH Royal Institute of Technology, Department of Applied Physics, Stockholm, Sweden*

S1. Core-levels fitting parameters

	BE and (FWHM) evolution of Sr 3d 5/2		BE and (FWHM evolution of Mg 2p	
	Grounded	Biased (+30V)	Grounded	Biased (+30V
**UHV**	133.84 (1.65)	133.95 (1.82)	50.02 (2.21)	50.01 (1.98)
	135.11 (1.65)			
**1 mbar H2O**	133.86 (1.97)	133.71 (1.6)	50.03 (1.81)	50.03 (1.66
**0,5 mbar H2O + 0,1 pyridine**	133.65 (1.7)	133.54 (1.7)	50.01 (1.61)	50.01 (1.62)

Table S1. Sr 3d and Mg 2p fitting parameter at hv=750 eV under grounded and biased condition

	hv=750 Grounded		hv=750 biased (+30V)
	O Bulk BE (FWHM)	H_2O Gas Phase BE (FWHM)	O Bulk BE (FWHM)
**UHV**	532.13 (2.78)	-	531.58 (2.5)
**0.5 mbar H_2O**	532.37 (2.71)	535.28 (1.2)	532.31 (2.7)
**0,5 mbar H_2O + 0,1 pyridine**	532.38 (2.49)	535.36 (0.99)	532.32 (2.7)
**0,5 mbar H_2O + 0,3 pyridine**	532.35 (2.49)	535.54 (0.93)	532.28 (2.5)
**0,5 mbar H_2O + 0,5 pyridine**	532.31 (2.22)	535.38 (0.89)	532.30 (2.48)
**After Pumping**	532.34 (2.89)	-	532.32 (2.94)

Table S2. O 1s fitting parameter at hv=750 eV fitting parameter under grounded condition

	hv=750 Grounded				hv=750 biased (+30V)	
	Pyr (ads)	Pyr gas phase	Pyr H-bond	N_2 gas phase	Pyr (ads)	Pyr-H bond
**0,5 mbar H2O + 0,1 pyridine**	**399.46 (1.85)**	**400.41 (0.92)**	**401.53 (1.85)**	**405.27 (0.8)**	**399.36 (1.94)**	**401.55 (1.94)**
**0,5 mbar H2O + 0,3 pyridine**	**399.46 (1.56)**	**400.46 (0.94)**	**401.57 (1.56)**	**-**	**399.42 (1.82)**	**401.51 (1.81)**
**0,5 mbar H2O + 0,5 pyridine**	**399.43 (1.49)**	**400.39 (1.85)**	**401.61 (1.49)**	**-**	**399.43 (1.85)**	**401.53 (1.85)**
**After Pumping**	**399.46 (2.31)**	**-**	**401.56 (2.31)**	**-**	**399.47 (2.28)**	**401.57 (2.28)**

Table S3. N 1s fitting parameter at hv=750 eV in grounded and biased condition

	hv=450 Grounded			
	Pyr (ads)	Pyr gas phase	Pyr H-bond	N_2 gas phase
**0,5 mbar H2O + 0,1 pyridine**	**399.5 (1.68)**	**400.35 (0.95)**	**401.3 (1.68**	**405.47 (0.83)**
**0,5 mbar H2O + 0,3 pyridine**	**399.55 (0.88)**	**400.52 (0.89)**	**401.66 (0.88)**	**-**
**0,5 mbar H2O + 0,5 pyridine**	**399.56 (0.98)**	**400.56 (0.81)**	**401.65 (0.98)**	**-**

After Pumping	399.54 (2.27)	-	401.54 (2.2)	-

Table S4. N 1s fitting parameter at hv=450 eV in grounded condition

	C-C BE (FWHM) eV	COC BE (FWHM) eV	C=O BE (FWHM) eV	CO_2 BE (FWHM) eV
UHV	284.84 (1.93)	286.48 (1.93)	288.41 (1.93)	-
0.5 mbar H2O	284.84 (1.67)	286.36 (1.67)	288.03 (1.67)	-
0,5 mbar H2O + 0,1 pyridine	284.86 (1.64)	286.37 (1.64)	287.98 (1.64)	-
0,5 mbar H2O + 0,3 pyridine	284.90 (1.63)	286.18 (1.63)	287.75 (1.63)	292.25 (1.63)
0,5 mbar H2O + 0,5 pyridine	284.94 (1.9)	286.51 (1.9)	287.74 (1.9)	292.23 (1.87)
After Pumping	285.00 (1.81)	286.64 (1.81)	288.15 (1.81)	-

Table S5. C 1s fitting parameter at hv=750 eV in biased condition

S2. Differential charging and its elimination

Clays are insulating materials. Under irradiation by the X-ray beam, the holes created by the photoemission process cannot be replenished by electrons provided by the ground. Therefore, the material becomes positively charged, and any reference with the analyzer Fermi level is lost.[1] Moreover differential charging can be observed as some parts of are better connected to the ground than others, and therefore charging is not uniform, laterally

and in-depth. To eliminate charging we use an original procedure. We bias the sample positively (+30 V) with respect to the analyzer and the chamber walls that are both grounded. Positive biasing leads to an increase in the flow of stray negative species produced by the X-ray beam in the analysis chamber (electrons in UHV, electrons and anions in NAP conditions). This is a self-compensating flood gun effect, superior to usual flood guns as overcompensation (i.e. a negative charging of the surface) never occurs. The elimination of differential charging in the XPS probed layer can be monitored by minimizing the full-width at half maximum (FWHM) of the core-levels, especially Mg 2p, as this species nested at the center of phyllosilicate lath is not affected by chemical changes occurring at the surfaces.

We find that a bias of +30 V is optimal. At hν=450 eV and with a pass energy of 50 V, biasing is impossible for the N 1s spectrum, due to a reduction of the photoelectron kinetic energy to a too low value of ~20 eV. In all cases, the binding energies given here are corrected from the bias.

The Sr 3d and Mg 2p spectra of the grounded sample and biased sample are given in Figure S1 (a) and (b), respectively. They exemplify the beneficial effects of pressure increase (NAP conditions) and positive biasing. The corresponding full-width at half maximum (FWHM) of Mg 2p are given in Figure S1. Differential charging is particularly acute in UHV conditions, see Figure S1 (a), bottom curve, for the grounded sample. The Sr 3d spectrum is fitted with *two* doublets, separated by 1.28 eV suggesting that two different chemical environments exist for the Sr^{2+} ion (for Sr 3d fitting parameters see section S4). In fact, this is only apparent, as once the +30 V bias is applied, Figure S1(b), the spectrum can be fitted with a *single* doublet. The Mg 2p FWHM of the UHV spectrum also changes from 2.21 eV to 1.99 eV when the bias is applied.

The mere increase of pressure improves the spectral resolution when the sample is grounded, as

shown in Figure S1 (a) and S1 (b). The Mg 2p FWHM is 1.81 eV under 0.5 mbar of H_2O and 1.61 eV under a pressure of 0.5 mbar of water plus 0.1 mbar of pyridine. However, the conjunction of both NAP conditions and sample biasing leads to optimal conditions for eliminating differential charging, as the Mg 2p FWHM is 1.66 eV under 0.5 mbar of water. Under 0.5 mbar of water plus 0.1 mbar of pyridine, the FWHM is 1.61 eV both in biased and non-biased conditions.

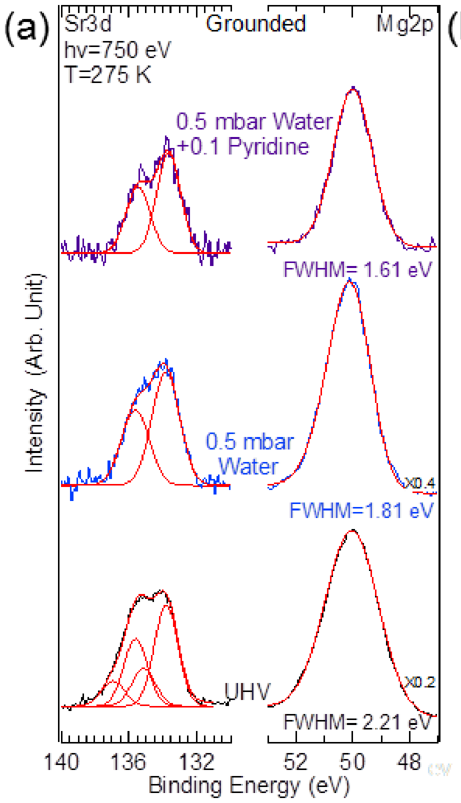

Figure S1: (a) Grounded sample. Differential charging affects less the Mg 2p and Sr 3d peak when the pressure increases. (b) biasing the sample (+30 V) leads to the reduction of differential charging already in UHV. The Mg 2p FWHM is indicated in the figure. The spectra are all aligned to the minimal binding energy of Mg 2p (measured with respect to the gold fermi level), i.e. 50.00 eV

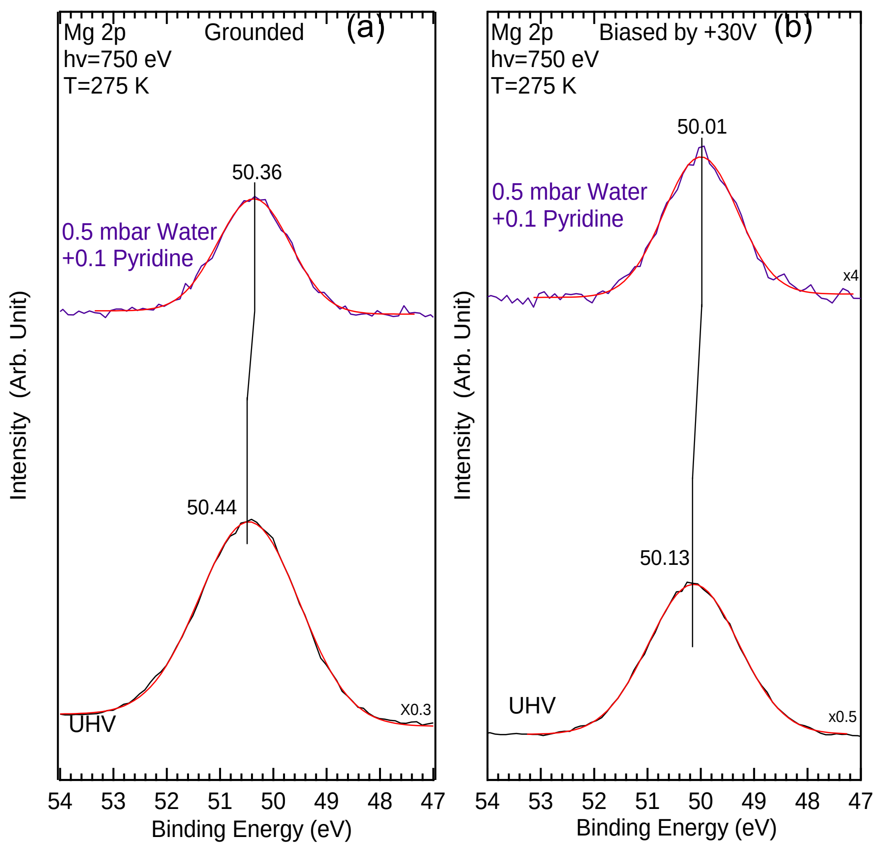

Figure S2: (a) Mg 2p peak position as a function of pressure, the sample being grounded. (b) same as (a) but in biased conditions (+ 30 eV is subtracted to the apparent binding energy). The Binding energies are referenced to the gold substrate Fermi level. Note that the compensation for charging is better in biased conditions. The presence of the gas phase still improves compensation as the Mg 2p peak reaches its minimum binding energy value of 50.01 eV.

Not only does the attenuation of differential charging decrease the FWHM of Mg 2p, but also the binding energy measured with respect to the gold substrate Fermi level decreases. For the biased spectra, the effect of increasing the pressure is shown in Figure S2 (b). When the sample is grounded, Figure S2 (a), the apparent binding energy of the Mg 2p spectrum stabilizes at 50.40 eV

(0.5 mbar of water plus 0.1 mbar of pyridine). In biased conditions, under pressures greater than 0.5 mbar, the position in binding energy of Mg 2p stabilizes at 50.00 eV, a binding energy lower by ~0.4 eV than in non-biased condition. The spectra shown in the main paper are all aligned to have the Mg 2p peak centered at 50.00 eV, and the O 1s centroid at 532.32 eV (used when Mg 2p spectra have a too weak intensity).

Taking these Mg 2p and O 1s binding energies as references, the C 1s the main component of contamination carbon (aliphatic carbon) is at 284.9±0.1 eV in UHV conditions, as expected for a compensated sample.[2] When pyridine is adsorbed in the clay, the carbon signal is dominated by that of the molecule, and we find that the peak maximum is at 284.80 eV. Indeed, adsorbed pyridine (e.g. on metals) shows a main C 1s peak at 284.8 (C=C component) and another one at 286 eV (CN component).[3,4]

S3. Can we distinguish surface Sr^{2+} from interlayer Sr^{2+} in UHV conditions?

The depth profiling (Figure S3) shows that while the Mg 2p FWHM is constant at 2 eV, the FWHM of Sr 3d increases from 1.72 eV (surface sensitive conditions, imfp of ~ 1.2nm) at hv=450 eV to 2.04 eV (more bulk condition, imfp of ~2.5 nm) at hv=1050 when fitted with a single doublet. Vertical differential charging is excluded as the FWHM of Mg 2p remains constant. However the situation is different for the counterion. Its binding energy may be affected by an initial state effect, related to different electrostatic potential energies at the open surface and within the interlayer. Final state effects may be also expected, as the relaxation energy[5] of a surface Sr^{2+} may be also different from that of an interlayer Sr^{2+} as it lies at the interface between an oxide and the vacuum, and is not sandwiched between two phyllosilicate sheets. However, Figure S3 shows that The broadening of the Sr 3d doublet due to the different chemical environments is so small that it

cannot be resolved experimentally.

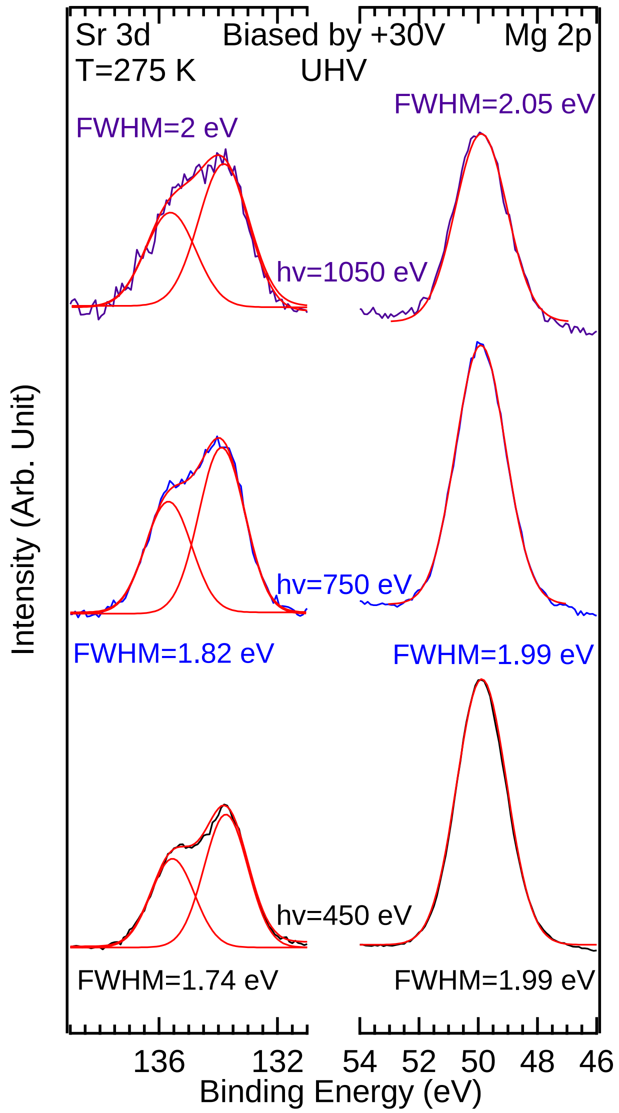

Figure S3: Evolution of the FWHM of Mg 2p and Sr 3d with different excitation energy (450, 750 and 1050 eV) under UHV condition. Note that the FWHM of the Sr 3d doublet increases while the photoelectron kinetic energy (and hence the probing depth) increase, while the FWHM of Mg 2p remains constant. All spectra are aligned with respect to the Mg 2p peak maximum at 50.00 eV.

S4. Electrostatic potential energy variation between the center of the clay layer and the counter ion plane

To estimate the electric field value in the clay structure, we consider three infinite successive parallel planes, with charge densities of $+\sigma/2$, $-\sigma$, and $+\sigma/2$, plunged into a dielectric medium of permittivity ε. d is the distance between the between the $-\sigma$ plane and the $+\sigma/2$ ones. By application of Gauss' law and of the superposition principle, the field is zero outside the three planes, equal to $\sigma/2\varepsilon$ between the $+\sigma/2$ plane and the $-\sigma$ one and equal to $-\sigma/2\varepsilon$ between the $-\sigma$ plane *and* $+\sigma/2$ one. Between the central $-\sigma$ plane and the $+\sigma/2$ ones, the electrostatic potential energy varies by $\Delta qV = -e\sigma d/2\varepsilon$, where e is the elementary charge This 3-plane unit cell can be used to build larger tactoids comprising N such unit cells. The chosen cell, and the boundary conditions, it involves are such that the divergence of the electrostatic energy with increasing N is avoided, as it occurs when the stacking of N $+\sigma/-\sigma$ alternately charged planes is considered.[6]

Each unit cell (0.524×0.909 nm^2) contains a charge of 0.8 e, therefore $|\sigma| = 0.34$ C.m^{-2}. The permittivity ε is $\varepsilon_0\varepsilon_r$, where ε_0 is the vacuum dielectric constant and ε_r is the relative dielectric constant. For the 0W and 1W hydration states, ε_r is estimated to be approximately 5 and 7, respectively.[7] In hydroxyhectorite the negatively charged plane is centered in the octahedral layer. Therefore, taking d between 0.38 nm (half a sheet width) and 1.02 nm (the distance between the middle of the sheet and the middle of the interlayer in the 1W state) and ε_r=7 one finds that $|\Delta qV|$ is between 1.0 and 2.8 eV. therefore, valence holes created in the central layer of the phyllosilicate sheet see a large energy barrier when move to the intersheet region where the cations are positioned. On the other hand, the valence electrons are pushed to the cations.

S5. Insertion of pyridine molecules in 1W Sr^{2+}-hydroxyhectorite

Let us consider the adsorption at the surface of the sheets or in the interlayer. Given the charge of the unit cell (0.8e) and the basal plane unit cell dimension of hectorite (0.524 nm × 0.909 nm[8]), there is an average free area around each strontium ion of 1.21 nm^2 (corresponding to an average distance between Sr^{2+} ions of 1.1 nm). This corresponds to ~5 ditrigonal cavities per intercalated ion. Assuming that at RH=7% the water molecules are all around the Sr^{2+} (about 4 for the 1W state),[9] there is room between the cations for the pyridine molecule, of diameter ~0.5 nm, to physisorb on siloxanes or to lock in the unoccupied ditrigonal holes (DFT calculations of pyridine interaction with the central HO^- in the Sr^{2+} free cavities lack).

We can get an estimate of the maximal pyridine insertion, based on the volume occupied by the hydrated Sr^{2+}/pyridine complex (assuming a basal spacing increase of 1.2 nm) and on that occupied by the pyridine molecule itself. The average free area around one strontium ion is 1.21 nm^2. In the model given in Refs. [10] and [11] the basal spacing increases by 1.2 nm (swelling due to water and pyridine), thus the free space around the strontium ion is 1.45 nm^3. Now the dimensions of the complex between the hydrated Sr^{2+} and pyridine can be estimated to be 1.2 nm (height) ×0.9 nm (width) ×0.26 nm (depth, i.e. the size of the Sr^{2+} solvation sphere[12,13]). The complex occupies a volume of 0.28 nm^3. Consequently, of the 1.45 nm^3 available space, 0.28 nm^3 are occupied by the complex, leaving 1.17 nm^3 free for "non H-bonded" pyridine molecules. Pyridine is a planar molecule with a diameter of 0.5 nm, whose minimal occupation volume can be estimated to be 0.063 nm^3 taking a face-to-face π-π stacking of 0.32 nm. This means that a maximum of 19 "non H-bonded" pyridine molecules per strontium ion can be inserted in the available space. Therefore, the maximum non "H-bonded" to "H-bonded" ratio is 19/4, that is 4.75.

A noticeable effect of pyridine adsorption onto the clay (at the surface and within the interlayer) is the strong damping of the Sr 3d and Mg 2p signals when pyridine partial pressures are greater than or equal to 0.3 mbar, as shown in Figure S4. See also the damping of the condensed-phase O 1s spectra in Figure 2 of the main paper.

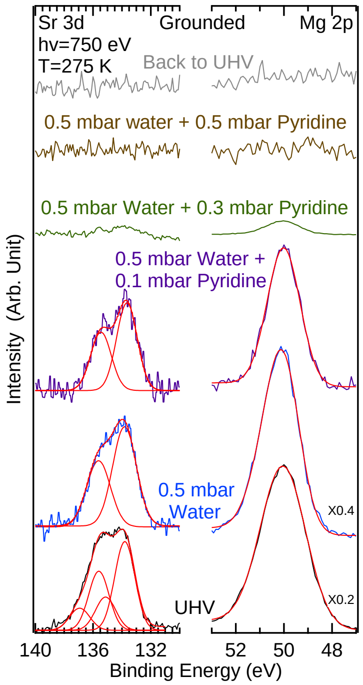

Figure S4. The Sr 3d and Mg 2p regions, for pyridine partial pressures between 0.1 and 0.3 mbar.

S6. Ionization cross sections at hν=450 and hν=750 eV

In order to discuss the *eh* pair generation by the soft X-ray beam, we first calculate the total cross sections corresponding to the $Sr_{0.4}Mg_{5.2}Li_{0.8}Si_{8.0}O_{20}(OH)_4$ formula at hν=450 eV and hν=750 eV, see Table S6. Note that both are very close. At hν=750 eV, the O 1s core-level is ionized with a

high cross-section, but the metal atom contribution is less than at hν=450 eV. We give also the total cross-sections for the pyridine molecule. As at hν=750 eV the energy distances from the C and N K edges are greater than at 450 eV, the total cross-section is smaller.

Orbital	Cross-sections in Mbarn at *hν*=450 eV (cross section for a unit cell)	Cross-sections in Mbarn at *hν*=750 eV (cross section for a unit cell)
O 2p	0.015 (0.36)	0.0028 (0.067)
O 2s	0.036 (0.864)	0.015 (0.36)
O 1s	-	0.24 (5.76)
Si 3s	0.013 (0.10)	0.0047 (0.038)
Si 3p	0.006 (0.048)	0.0015 (0.012)
Si 2p	0.4 (3.2)	0.093 (0.744)
Si 2s	0.14 (1.1)	0.055 (0.44)
Mg 3s	0.005 (0.026)	0.0018 (0.01)
Mg 2p	0.18 (0.94)	0.041 (0.21)
Mg 2s	0.1 (0.52)	0.036 (0.19)
Li 2s	0.0004 (0.0003)	$8\ 10^{-5}$ ($6\ 10^{-5}$)
Li 1s	0.03 (0.024)	0.006 (0.005)
Sr 5s	0.0027 (0.0011)	0.0011 (0.0005)
Sr 4p	0.09 (0.036)	0.039 (0.016)
Sr 4s	0.03 (0.012)	0.013 (0.005)
Sr 3d	1.9 (0.76)	0.53 (0.21)
Sr 3p	0.59 (0.24)	0.31 (0.12)
Sr 3s	0.15 (0.06)	0.077 (0.031)

Total cross-section corresponding to $Sr_{0.4}Mg_{5.2}Li_{0.8}Si_{8.0}O_{20}(OH)_4$ formula	8.29	8.22
N 2p	0.0055 (0.0055)	0.001 (0.0055)
N 2s	0.0245 (0.0245)	0.007 (0.007)
N 1s	0.545 (0.545)	0.0155 (0.0155)
C 2p	0.0015 (0.0075)	0.0003 (0.0015)
C 2s	0.015 (0.075)	0.004 (0.02)
C 1s	0.35 (1.75)	0.093 (0.465)
Pyridine total cross section corresponding to C_5H_5N formula	2.4	0.50

Table S6. Photoionization cross-sections, from Ref.[14] *of the clay constituent atoms (for numbers between brackets, the stoichiometry* $Sr_{0.4}Mg_{5.2}Li_{0.8}Si_{8.0}O_{20}(OH)_4$ *is accounted for). Photoionization cross-sections of the pyridine constituent atoms (for numbers between brackets the stoichiometry of the molecule is accounted for).*

S7. Hole-electron pair formation and irradiation doses

According to Cazaux,[15] the number of *eh* pairs *n(eh)* produced by one (absorbed) photon of energy $h\nu$ and having excited a given core-level is:

$$n(eh) = \frac{KE_{PE}}{E(eh)} + a_{Auger}\frac{KE_{Auger}}{E(eh)} \quad (1)$$

where *E(eh*) the energy for creating a *eh* pair, KE_{PE} the kinetic energy of the photoelectron, and KE_{Auger} the kinetic energy of the Auger electron, and a_{Auger} the probability of filling the core-

hole via an Auger decay. a_{Auger} is close to one for the light elements of the 2nd period, C, N, O (KLL transitions) and of the 3rd period, Mg, Si (LVV transitions). With $KE_{PE} = h\nu - BE$, $KE_{Auger} \approx BE$, and $a_{Auger} \approx 1$ (where BE is core-level binding energy) one gets:

$$n(eh) \approx \frac{h\nu}{E(eh)} \quad (2)$$

Concerning the heavier element Sr (5th period) the higher binding energy level that is excited is the M_1 level (3s). Its a_{Auger} decay probability is also close to one.

E(eh) is twice to three times the material gap. It is 17 eV for SiO_2,[15] for a band gap of 9.3 eV.[16] Given that the band gap of hectorite is 4.0-4.5 eV,[17] we will take *E(eh)* equal to 9 eV in our calculations. The pair generation factor per unit volume and per unit time $g(eh)$ may also be expressed as a function of $\Phi_0$the incident photon flux, and μ the linear absorption coefficient of the specimen for the X-ray photons of interest as:

$$g(eh) = \frac{h\nu\Phi_0\mu}{E(eh)} \text{ (in cm}^{-3}\text{ s}^{-1}\text{)}$$

The mass absorption coefficient μ* in dry Sr^{2+}-hydroxyhectorite of composition $Sr_{0.4}Mg_{5.2}Li_{0.8}Si_{8.0}O_{20}(OH)_4$ can be calculated from the mass absorption coefficients of its atomic constituents. Then the linear mass absorption knowing the mass density of Sr^{2+}- hydroxyhectorite (~2.5 g/cm^3) as $\mu = \mu^* \times \rho$

Element	Atomic mass	μ* at hv=750ev[18]	μ* at hv=450ev[18]
Si	28.09	3514 cm²/g	12950 cm²/g
Mg	24.31	1936 cm²/g	7655 cm²/g
Li	6.94	542 cm²/g	2544 cm²/g
Sr	87.62	6643 cm²/g	18288 cm²/g
O	15.999	9673 cm²/g	1778 cm²/g
H	1.008	17,6 cm²/g	95 cm²/g
**Clay**	779.74	**6393 cm²/g**	**6689 cm²/g**
**μ**	-	**15981 cm^{-1}**	**16723 cm^{-1}**

The mass absorption coefficient have been gathered from http://henke.lbl.gov/optical_constants/

At $h\nu$=750 eV, Φ_0= 4×10^{16} photon/s/cm^2. With $E(eh)$= 4.5 eV[17] and μ=15981 cm^{-1}, we find $g(eh)$ =2.6×10^{22} cm^{-3} s^{-1}. The radiation dose rate $\dot{D}$ received at the sample surface can be estimated with the following formula $\dot{D}$=Φ_0hν μ* , i.e. 3.1×10^7 Gy/s. As a typical N 1s spectrum is taken within a time t_i of 25 s, the irradiation dose D is 7.68×10^8 Gy, so 14×10^{23} *eh* pairs per cm^{-3} are generated during the acquisition.

At $h\nu$=450 eV, Φ_0= 2×10^{16} photon/s/cm^2, $E(eh)$=4.5 eV and μ=16723 cm^{-1}, we find $g(eh)$ =8.5×10^{21} cm^{-3} s^{-1}. A typical N 1s spectrum is still taken within a time t_i of 25 second, so 4.4×10^{23} *eh* pairs per cm^{-3} are generated during the acquisition. The radiation dose rate $\dot{D}$ received at the sample surface is then 10^7 Gy/s. During the acquisition of one spectrum (t_i =25 s), the dose D is 2.4×10^8 Gy.

Considering that we are working in the so-called top-up mode with 28 ps wide pulses each separated by 2.84 ns (352 MHz),[19] each pulse emits a radiation dose of 8.81×10^{-2} (2.76×10^{-2}) Gy at hν=750 eV (450 eV).

Let us now compare with the pulsed electron irradiation experiments of Refs. [20] and [21]. 10 MeV electron pulses of 10 ns duration at a repetition rate of 2 Hz are used. The dose per pulse is 30 Gy, corresponding to a dose rate of 3×10^9 Gy/s, two orders of magnitude greater than in the present case. However, typical doses received by the samples are 1.4×10^5 Gy for the whole experiment, three orders of magnitude smaller than during a typical XPS scan of 25 s.

S8. C 1s spectrum of the grounded sample

As shown in Figure S5, the C 1s peak of gas phase pyridine is found at a binding energy of 286.6 eV (maximum). The ionization energy (with respect to vacuum level) of the o-, m- and p- carbons are at 291.1, 290.5 and 290.8 eV respectively.[22] Gas phase C 1s ionization energies of typical small C_xH_y molecules CH_4, C_2H_2, C_2H_4 and C_2H_6 are measured at 290.9, 291.2, 290.7 and 290.76 eV, respectively.[23] Therefore, any small non nitrogenous C_xH_y molecule going into the gas phase after pyridine dissociation cannot be distinguished from gaseous pyridine.

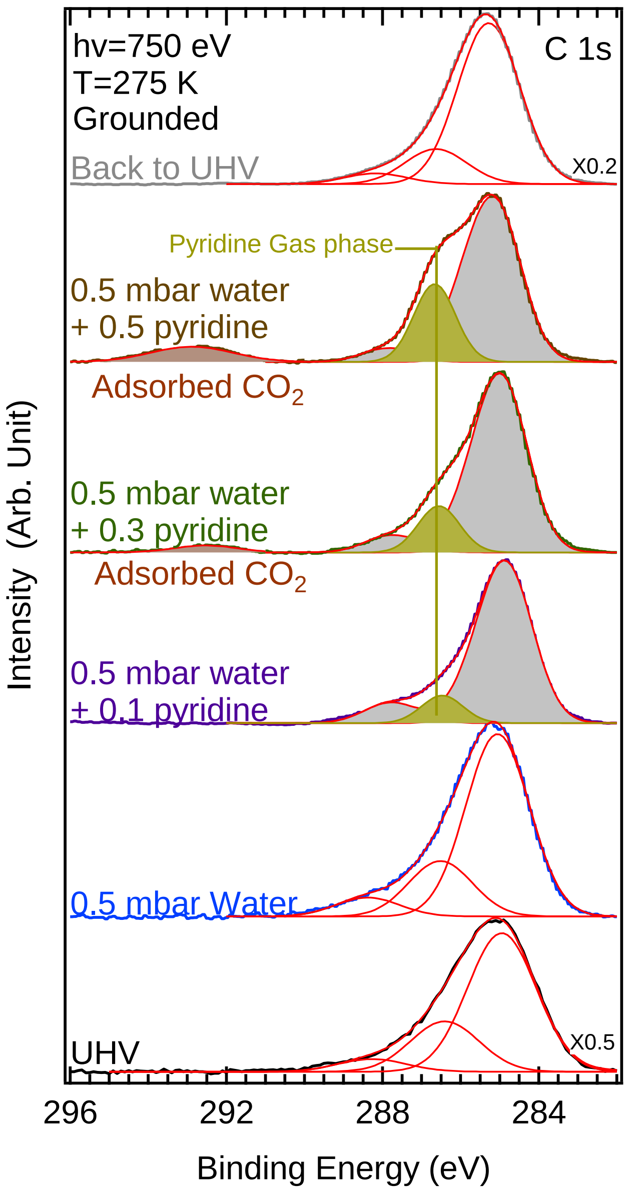

Figure S5. C 1s spectrum measured at hν=750 eV (grounded sample).

References

1. Egelhoff, W. F. Core-level binding-energy shifts at surfaces and in solids. *Surf. Sci. Rep.* **6,** 253–415 (1987).

2. Barr, T. L. Nature of the use of adventitious carbon as a binding energy standard. *J. Vac. Sci. Technol. A Vacuum, Surfaces, Film.* **13,** 1239 (1995).

3. Essafti, A., Ijdiyaou, Y. & Azizan, M. XPS study of amorphous carbon nitride (a-C : N) thin films deposited by reactive RF sputtering. **90,** 1420–1423 (2006).

4. Anna, E. D. *et al.* Study of C Z N binding states in carbon nitride ® lms deposited by reactive XeCl laser ablation. **347,** 72–77 (1999).

5. Egelhoff, W. F. Core-level binding-energy shift at surface and in solids. *Surf. Sci. Rep.* **6,** 253–415 (1987).

6. Goniakowski, J., Finocchi, F. & Noguera, C. Polarity of oxide surfaces and nanostructures. *Reports Prog. Phys.* **71,** 16501 (2008).

7. Laird, D. Model for crystalline swelling of 2: 1 phyllosilicates. *Clays Clay Miner.* **44,** 553–559 (1996).

8. Breu, J., Seidl, W. & Stoll, A. Fehlordnung bei Smectiten in Abhängigkeit vom Zwischenschichtkation. *Zeitschrift für Anorg. und Allg. Chemie* **629,** 503–515 (2003).

9. Young, D. a. & Smith, D. E. Simulations of Clay Mineral Swelling and Hydration Dependence upon Interlayer Ion Size and Charge. *J. Phys. Chem. B* **104,** 9163–9170 (2000).

10. Farmer, V. C. & Mortland, M. M. An infrared study of the co-ordination of pyridine and water to exchangeable cations in montmorillonite and saponite. *J. Chem. Soc. A Inorganic, Phys. Theor.* 344 (1966). doi:10.1039/j19660000344

11. Ukrainczyk, L. & Smith, K. A. Solid State 15 N NMR Study of Pyridine Adsorption on Clay Minerals. *Environ. Sci. Technol.* **30,** 3167–3176 (1996).

12. Tommaso, D. Di & De Leeuw, N. H. First Principles Simulations of the Structural and Dynamical Properties of Hydrated Metal Ions Me 2 þ and Solvated Metal Carbonates (Me = Ca , Mg , and Sr). (2010). doi:10.1021/cg100055p

13. Persson, I. Hydrated metal ions in aqueous solution : How regular are their structures ? *Pure Appl. Chem.* **82,** 1901–1917 (2010).

14. Yeh, J. J. & Lindau, I. Atomic Subshell Photoionization Cross Sections and Asymmetry Parameters: 1 < Z <103. *At. Data Nucl. Data Tables* **32,** 1–155 (1985).

15. Cazaux, J. A physical approach to the radiation damage mechanisms induced by X-rays in X-ray microscopy and related techniques. *J. Microsc.* **188,** 106–124 (1997).

16. Weinberg, Z. A., Rubloff, G. W. & Bassous, E. Transmission, photoconductivity, and the

experimental band gap of thermally grown SiO2 films. *Phys. Rev. B* **19,** 3107–3117 (1979).

17. Horwath, W. & Liang, Y. L. *Variations of Chemical Composition and Band Gap Energies in Hectorite and Montmorillonite Clay Minerals on Sub-Micron Length Scales. Final Report. Kearney Foundation of Soil Science*. (2011).

18. Henke, B. L., Gullikson, E. M. & Davis, J. C. X-Ray Interactions Photoabsoption, Scattering, Transmission and reflection at E=50-30,000 eV, Z=1-92. *Data Nucl. Data Tables* **54,** 181 (1993).

19. Soleil. Storage ring filling patterns. *Mag. SOLEIL Synchrotron* 12–14 (2013).

20. Fourdrin, C. *et al.* Water radiolysis in exchanged-montmorillonites: the H2 production mechanisms. *Environ. Sci. Technol.* **47,** 9530–7 (2013).

21. Lainé, M. *et al.* Reaction mechanisms in swelling clays under ionizing radiation: influence of the water amount and of the nature of the clay mineral. *RSC Adv.* **7,** 526–534 (2017).

22. Kolczewski, C. *et al.* Detailed study of pyridine at the C 1s and N 1s ionization thresholds: The influence of the vibrational fine structure. *J. Chem. Phys.* **115,** 6426–6437 (2001).

23. Jolly, W. L., Bomben, K. D. & Eyermann, C. J. Core-electron binding energies for gaseous atoms and molecules. *At. Data Nucl. Data Tables* **31,** 433–493 (1984).
